# Secondhand smoke knowledge, sources of information, and associated factors among hospital staff

**DOI:** 10.1371/journal.pone.0210981

**Published:** 2019-01-22

**Authors:** Sae Rom Lee, A-ra Cho, Sang Yeoup Lee, Young Hye Cho, Eun Ju Park, Yun Jin Kim, Jeong Gyu Lee

**Affiliations:** 1 Family Medicine Clinic, and Research Institute of Convergence of Biomedical Science and Technology, Pusan National University Yangsan Hospital, Yangsan, Republic of Korea; 2 Department of Medical Education, Pusan National University School of Medicine, Yangsan, Republic of Korea; 3 Department of Family Medicine, Pusan National University School of Medicine, Yangsan, Republic of Korea; University of Westminster, UNITED KINGDOM

## Abstract

**Purpose:**

To evaluate knowledge of secondhand smoke (SHS) risks, sources of information, and associated factors and behaviors among hospital staff.

**Methods:**

We conducted a cross-sectional survey using a 40-item self-administered questionnaire among 328 employees at a university hospital. The questions on representative diseases related to SHS were used to measure the degree of knowledge of SHS. Multiple regression analysis was used to determine the correlation between SHS knowledge scores and variables.

**Results:**

Females had better SHS knowledge scores than males, regardless of smoking status (p<0.05). SHS knowledge was positively correlated with cessation education in males, non-smokers, and the total sample (β = 3.950, 2.356, and 2.684, respectively, p<0.05). It was correlated with the experience of any SHS exposure-related symptoms in males, non-smokers, and the total sample (β = 3.950, 2.356, and 2.684, respectively, p<0.05) and discomfort when exposed to SHS in non-smokers and the total sample (β = 0.670 and 0.821, respectively, p<0.05).

**Conclusion:**

SHS knowledge is high among females, when hospital staff are educated about SHS risks, and when they have experienced any SHS exposure-related symptoms or felt uncomfortable when exposed to SHS. SHS risk education is an effective tool to increase SHS knowledge in hospital staff.

## Introduction

According to the World Health Organization (WHO), more than 600,000 people die of secondhand smoke (SHS) each year. SHS causes lung cancer, stroke, and coronary artery disease in adults and sudden infant death syndrome, respiratory disease, and lung dysfunction in children [1]. According to the WHO Framework Convention on Tobacco Control, many countries are making efforts to protect their citizens from the damage caused by exposure to tobacco smoke [2]. In order to prevent the harmful effects of SHS, since 2012, non-smoking areas in public places, such as public facilities, resting places, and restaurants, have continued to expand in South Korea. It is also important to inform both smokers and non-smokers about the risks of SHS. Previous studies have shown that smokers are more likely to quit smoking as their knowledge of SHS increases [3]; once they are aware of it risks, they avoid exposing adults and children around them to smoke [4]. Some studies have analyzed the factors affecting knowledge of SHS. In those studies, when evaluating knowledge of SHS, the study subjects were considered knowledgeable when they chose one of two response options—“know” or “know a little”—about SHS, or knew about some SHS-related illness. However, the degree of knowledge about SHS has not been measured more specifically and objectively [5, 6]. Also, few studies have analyzed the factors affecting the degree of knowledge of passive smoking. The objectives of this study were to: i) estimate smoking prevalence, ii) to evaluate their knowledge and attitudes towards SHS risks and iii) determine whether factors contribute to SHS knowledge among hospital staffs. We hypothesized that the degree of SHS knowledge would differ according to smoking status and gender, and that SHS knowledge scores might be linked to factors such as sources of information about SHS risks and experiences with SHS-related symptoms or discomfort when exposed to SHS in hospital staff.

## Material and methods

### Study subjects and design

This study, conducted from September 1, 2016 to February 14, 2018, involved 2,166 employees (434 physicians, 1,251 nurses, 123 administrative staff, 323 health service technicians, 35 pharmacists) working at a university hospital in Yangsan. Self-reporting questionnaires were distributed to each department. Of the 2,166 subjects, 340 (15.78%) responded to the survey. Among them, a total of 328 subjects completed all the questions required for data analysis. The study was approved by the Institutional Review Board of Pusan National University Yangsan Hospital (IRB No. 05-2016-063). Written informed consent was obtained from all subjects.

### Questionnaires

The questionnaires were composed of 40 questions, divided into three parts. In the first part, we examined the demographic characteristics including age, gender, family members, education, medical history, occupation, career, and alcohol history. In the second part, we evaluated smoking history, the Fagerstrom Test for Nicotine Dependence (FTND), smoking area, the experience of smoking cessation, smoking cessation plan, and reason for quitting smoking. In the third part, we asked about the perception of SHS health risks and the sources of information. A pilot study was conducted with 30 employees to determine specific sources of information before the start of this study. As a result, five information sources related to SHS health risks—acquaintances, smoking cessation education, public service ads, TV programs, and the news—were selected. In the fourth part, we asked eight questions to measure SHS knowledge, eight questions about SHS-related illnesses (lung cancer, heart disease, and cognitive deficits in adults and low birth weight, ear infection, heart attack, allergies, or asthma in children) to measure knowledge of SHS [7–9]. The respondents were asked to indicate on a five-point Likert scale to what degree they agreed with each statement: strongly agree (5), agree (4), neutral (3), disagree (2), and strongly disagree (1). In addition, we investigated whether the study subjects were exposed to SHS. If exposed, we determined where they were primarily exposed, how they felt when they were exposed, and how they coped. Finally, we asked their opinion about non-smoking areas in public places.

### Statistical analysis

The sample size was calculated using the StatCalc programme in Epi Info Version 7. The minimum number of questionnaires required to have a confidence interval of 95% with a margin of error of 5% among our 2,150 hospital staff was 327 [5]. Descriptive data were expressed as means (SDs) and percentages. Normally distributed data, as determined using the Shapiro-Wilk test, were expressed as means and SDs, whereas variables with a skewed distribution were reported as medians and ranges. Between-group baseline characteristic comparisons were performed using the two-sample t-test or Mann-Whitney U test for continuous variables or the chi-squared test for categorical variables. When asked about knowledge of SHS, “strongly agree” or “agree” were considered the correct answers, and the comparison of the correct answer rates was performed using the chi-squared test. Pearson’s correlation was performed to detect variables that were highly correlated with the total SHS score. To study the influence on SHS knowledge, multiple linear regression analyses were performed with age and FTND score as dependent variables. The multiple regression analysis included sex, cessation education as a source of information on SHS-related knowledge, uncomfortableness when exposed to SHS, and any SHS exposure symptom. These imposes a potential factor in relation to the non-normal distribution when determining the corresponding P-value. These were addressed by using logistic regression with sex, cessation education as a source of information on SHS-related knowledge, uncomfortableness when exposed to SHS, and any SHS exposure symptom as the dependent variables. Statistical analysis of the data was performed using SPSS version 21.0 for Windows (SPSS Inc, Chicago, USA). Statistical significance was accepted for p-values <0.05.

## Results

### General characteristics of subjects

Among the survey respondents, 40 (12.1%) were smokers and all smokers were male (p<0.001). The age distribution of smokers under 30 and in their 30s was similar, but most non-smokers were under 30. Among smokers, the proportion of heavy drinkers was more than twice that among non-smokers (p<0.001). Among smokers, the percentage of those who were below high school graduates was 15 times higher than that among non-smokers (p<0.001). All female participants were non-smokers and mostly nurses, so the distributions of the occupations of smokers and non-smokers were different. There were no differences in the year of service and family members (Table 1).

**Table 1:** Sociodemographic characteristics of subjects.

Variable	Smoker (n = 40)	Non-Smoker (n = 288)	p value^a^
Age			
<31	19 (47.5)	185 (64.2)	0.240
31–40	20 (50.0)	84 (29.2)	
>40	1 (2.5)	19 (6.6)	
Sex			
Men	40 (100.0)	63 (21.9)	**<0.001**
Women	0 (0.0)	225 (78.1)	
Education			
= <High school	6 (15.0)	3 (1.0)	**<0.001**
>High school	34 (85.0)	285 (99.0)	
Alcohol			
Binge drinking^b^	32 (80.0)	109 (37.8)	**<0.001**
Job			
Nurses	1 (2.5)	158 (54.9)	**<0.001**
Health care staffs	7 (17.5)	32 (11.1)	
Pharmacists	6 (15.0)	5 (1.7)	
Physicians	6 (15.0)	47 (16.3)	
Administrative staffs	10 (25.0)	28 (9.7)	
Manual workers	10 (25.0)	18 (6.3)	
Employment period (year)	5.6 ± 4.2	5.3 ± 4.3	0.520
Family	26 (65.0)	194 (67.4)	0.206
Spouse	11 (27.5)	75 (26.0)	0.259
Child	6 (15.0)	53 (18.4)	0.276
Subjective perception^c^	39 (97.5)	288 (100.0)	0.247

^a^By χ2 tests except employment period (two-sample *t*-test)

^b^More than five glasses/once

^c^When they answered “I know” to the question “Do you know about the harm of secondhand smoke?”

### SHS knowledge

With “strongly agree” and “agree” considered correct answers regarding SHS knowledge, 85.5% of the non-smokers and 63.8% of the smokers, respectively, answered correctly. The percentage of correct answers for lung cancer (p<0.001), heart disease (p = 0.020), and cognitive deficits (p = 0.033) in adults and allergies (p = 0.025) and asthma (p<0.001) in children among non-smoking males was higher than among smoking males, but there was no difference in the average number of items answered correctly. Comparing non-smokers among both males and females, the percentage of correct answers for heart disease (p = 0.044) in adults and ear infection (p<0.001), heart attack (p<0.001), allergy (p = 0.033), and asthma (p<0.001) in children and the total number of correct answers provided by females was higher (p<0.001) than that of non-smoking males. In a gender-based comparison, females had higher rates of correct answers (p = 0.022~p<0.001) than males (smokers and non-smokers) on all eight questions (p<0.001) (Table 2).

**Table 2:** Percentage of correct answers about SHS knowledge according to gender and smoking status.

Knowledge	Men			Women	p value^a^	p value^b^	p value^c^
	Smoker (n = 40)	Non-smoker (n = 63)	Total (n = 103)	Non-smoker (n = 225)			
SHS causes lung cancer.	25 (62.5)	58 (92.1)	83 (80.6)	216 (96.0)	**<0.001**	0.199	**<0.001**
SHS causes heart disease.	24 (60.0)	51 (81.0)	75 (72.8)	203 (90.2)	**0.020**	**0.044**	**<0.001**
SHS is associated with cognitive deficits	29 (72.5)	56 (88.9)	85 (82.5)	209 (92.9)	**0.033**	0.301	**0.004**
SHS causes low birth weight.	30 (75.0)	53 (84.1)	83 (80.6)	202 (89.0)	0.254	0.213	**0.022**
SHS causes ear infection in children.	23 (57.5)	29 (46.0)	52 (50.5)	160 (71.1)	0.257	**<0.001**	**<0.001**
SHS causes heart attack to children	24 (60.0)	35 (55.6)	59 (57.3)	180 (80.0)	0.657	**<0.001**	**<0.001**
SHS is associated with allergies in children.	22 (55.0)	48 (76.2)	70 (68.0)	196 (87.1)	**0.025**	**0.033**	**<0.001**
SHS is associated with asthma in children.	27 (67.5)	60 (95.2)	87 (84.5)	213 (91.5)	**<0.001**	0.857	**0.002**
Average of the total number of correct answers	5.10 ± 2.93	6.19 ± 1.91	5.77 ± 2.41	7.02 ± 1.71	0.160	**<0.001**	**<0.001**

Abbreviations: SHS, secondhand smoke

It is regarded as the correct answer if subjects choose “strongly agree” or “agree” from among the following options: strongly agree, agree, neutral, disagree, strongly disagree,

^a^Between smoking and non-smoking males,

^b^between non-smoking males and non-smoking females,

^c^between males and females by a chi-squared test

### Sources of SHS-related knowledge

**Fig 1:**
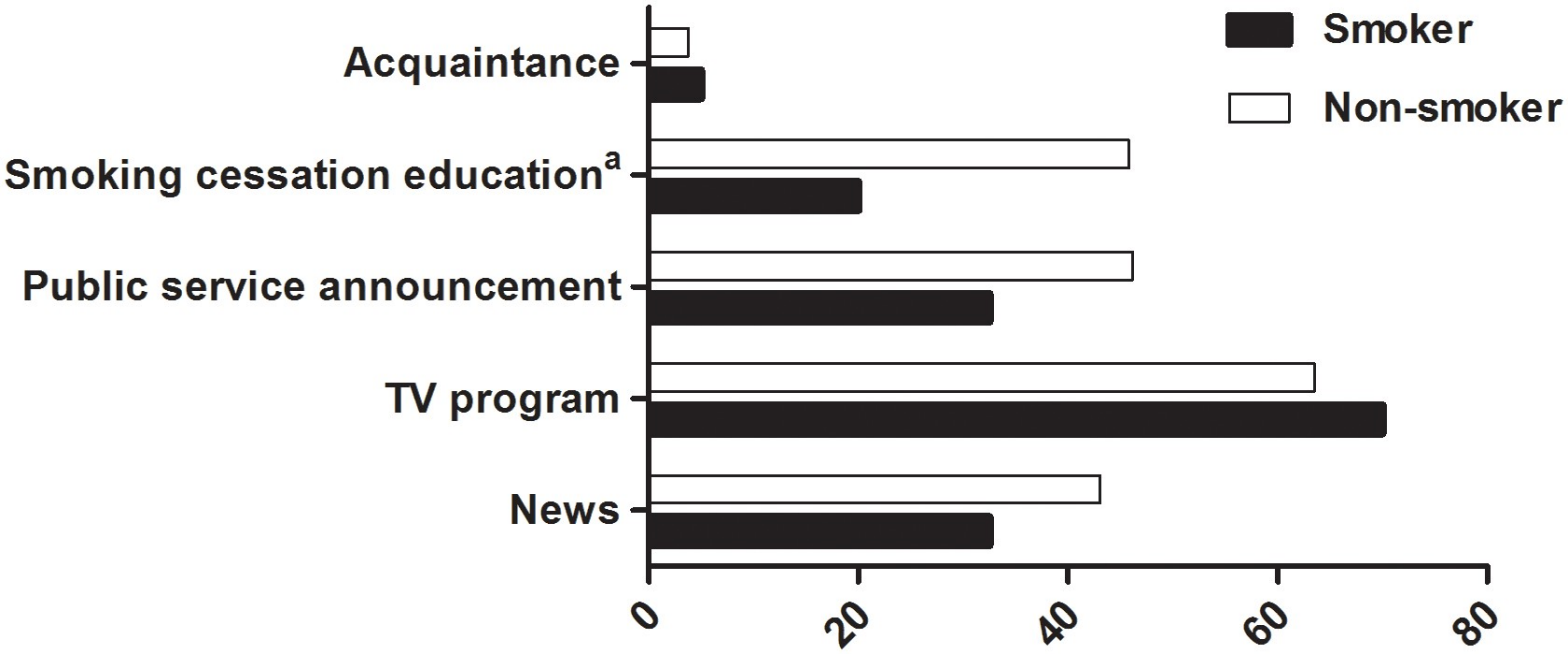
Sources of secondhand smoke-related knowledge. Data expressed as percent. ^a^p = 0.002 between smokers and non-smokers.

The most common source of SHS-related knowledge was TV programs. Next, non-smokers learned about SHS through smoking cessation education, while smokers received information through public service announcements or the news rather than smoking cessation education (p<0.05, Fig 1).

### Places of exposure to SHS

**Fig 2:**
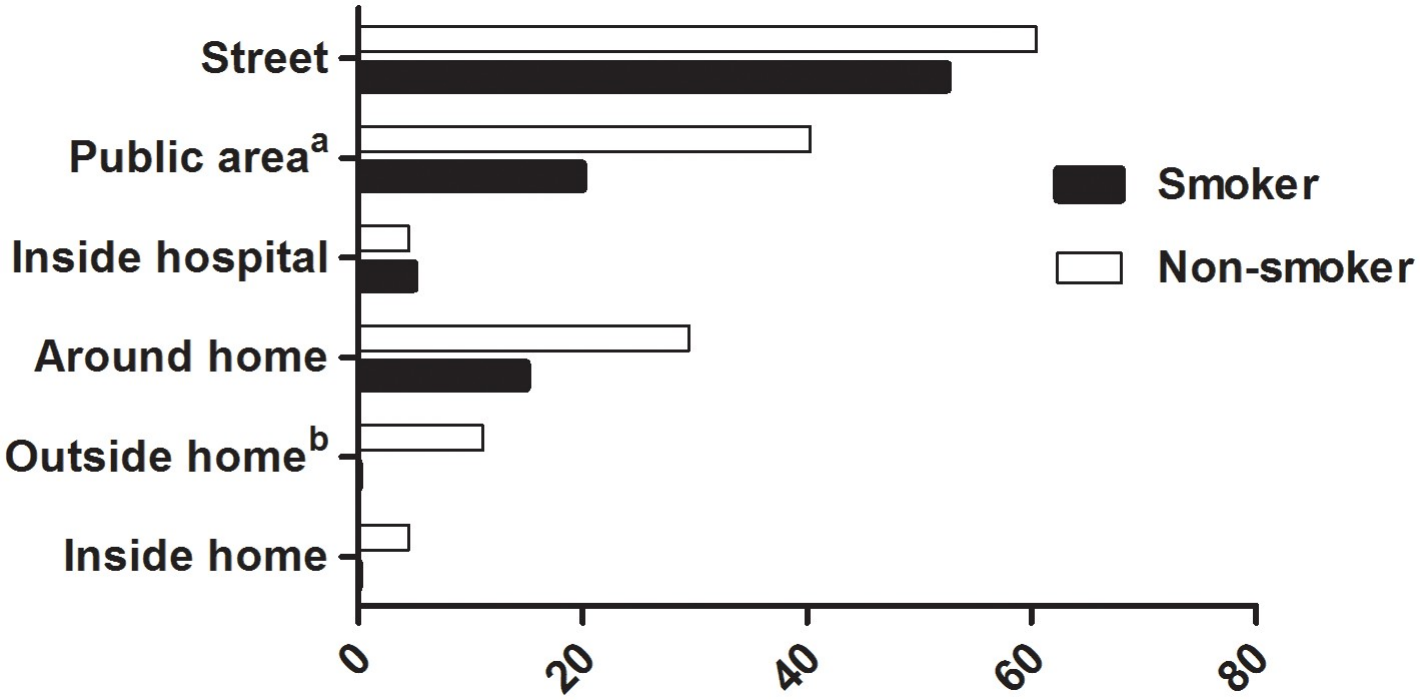
Places of exposure to secondhand smoke (%). Data expressed as percent. ^a^p = 0.014, ^b^p = 0.021 between smokers and non-smokers.

Both smokers and non-smokers answered that the most common place of SHS exposure was the street. Next were public spaces and areas around the hospital. None of the smokers responded that they had been exposed at home, while a few non-smokers reported exposure to SHS at home (Fig 2).

### Factors related to SHS knowledge

In males, the SHS knowledge score showed a positive correlation with smoking status (r = 0.211), number of packs smoked per day (r = 0.264), cessation education as a source of SHS-related knowledge (r = 0.344), discomfort when exposed to SHS (r = 0.218), irritation of the nose and eyes (r = 0.208), chest discomfort (r = 0.304), or any other symptom (r = 0.300) when exposed to SHS and a negative correlation with smoking year (r = -0.237), FTND score (r = -0.239), or TV programs as a source of SHS-related knowledge (r = -0.202). In the case of non-smokers, SHS knowledge was related to gender (r = 0.162), cessation education as a source of SHS-related knowledge (r = 0.210), discomfort when exposed to SHS (r = 0.173) and having respiratory (r = 0.135) or any other symptoms (r = 0.149) when exposed to SHS. In all respondents, Pearson’s correlation analyses showed that the SHS knowledge score had a positive correlation with gender (r = 0.255), smoking status (r = 0.247), cessation education as a source of SHS-related knowledge (r = 0.259), discomfort when exposed to SHS (r = 0.246), irritation of the nose and eyes (r = 0.142), respiratory symptoms (r = 0.193), chest discomfort (r = 0.125), or any other symptoms (r = 0.149) when exposed to SHS, while it had a negative correlation with age (r = -0.147), number of packs smoked per day (r = -0.246), smoking year (r = -0.233), and FTND score (r = -0.239) (Table 3).

**Table 3:** Factors related to SHS knowledge.

Variables	Men		Non-smoker		Total	
	r	p value	r	p value	r	p value
Age	-0.182	0.066	-0.111	0.061	**-0.147**	**0.007**
Sex (men 0, women 1)			**0.162**	**0.006**	**0.255**	**<0.001**
Smoker (Nonsmoker 0, Smoker 1)	**0.211**	**0.032**			**0.247**	**<0.001**
Smoking (pack/day)	**0.264**	**0.007**			**-0.246**	**<0.001**
Smoking (year)	**-0.237**	**0.016**			**-0.233**	**<0.001**
FTND score	**-0.239**	**0.015**			**-0.239**	**<0.001**
Sources of information on SHS-related knowledge						
Newspapers	0.003	0.978	-0.011	0.859	-0.015	0.786
TV programs	**-0.202**	**0.030**	0.010	0.870	-0.034	0.535
Public service announcement	0.058	0.561	0.044	0.461	0.057	0.303
Smoking cessation education	**0.344**	**<0.001**	**0.210**	**<0.001**	**0.259**	**<0.001**
Acquaintance	-0.084	0.397	-0.084	0.156	**-0.090**	**0.103**
Uncomfortableness when exposed to SHS^a^	**0.218**	**0.027**	**0.173**	**0.003**	**0.246**	**<0.001**
Symptoms of exposure to SHS						
Irritation of the nose and the eyes	**0.208**	**0.035**	0.073	0.218	**0.142**	**0.010**
Respiratory symptom	0.187	0.058	**0.135**	**0.022**	**0.193**	**<0.001**
Chest discomfort	**0.304**	**0.002**	0.106	0.073	**0.125**	**0.023**
Child respiratory	0.014	0.887	0.000	0.997	0.000	0.997
Any symptom	**0.300**	**0.002**	**0.149**	**0.011**	**0.149**	**0.011**

r = Pearson’s correlation coefficients

SHS, secondhand smoke; FTND, Fagerstrom Test for Nicotine Dependence

^a^strongly disagree 1, disagree 2, neutral, 3, agree 4, strongly agree 5

Multiple regression analyses showed that SHS knowledge was independently predicted by cessation education as a source of SHS-related knowledge (β = 3.656) and any SHS exposure symptom (β = 3.950) in males, and was independently predicted by gender (β = 1.335), cessation education as a source of SHS-related knowledge (β = 2.060), discomfort when exposed to SHS (β = 0.670), or any SHS exposure symptom (β = 2.356) in non-smokers. In all respondent, SHS knowledge was independently predicted by cessation education as a source of information on SHS-related knowledge (β = 2.253), discomfort when exposed to SHS (β = 0.260), or any SHS exposure symptom (β = 1.006) (Table 4).

**Table 4:** Multiple regression analysis for the prediction of SHS knowledge.

Independent variables	Men			Non smoker			Total		
	β	SE	p value	β	SE	p value	β	SE	p value
Sex (men 0, women 1)				**1.335**	**0.675**	**0.049**	1.107	0.660	0.094
Smoking (pack/day)	-0.091	0.167	0.586				-0.112	0.154	0.469
FTND score	0.001	0.470	0.986				0.065	0.436	0.881
Cessation education as a source of information on SHS-related knowledge (no 0, yes 1)	**3.656**	**1.103**	**0.001**	**2.060**	**0.545**	**<0.001**	**2.253**	**0.535**	**<0.001**
Uncomfortableness when exposed to SHS^a^	0.724	0.414	0.084	**0.670**	**0.273**	**0.015**	**0.821**	**0.260**	**0.002**
Any SHS exposure symptom (no 0, yes 1)	**3.950**	**1.224**	**0.002**	**2.356**	**0.841**	**0.006**	**2.684**	**1.006**	**<0.001**

β, regression coefficient; SE, standard error; SHS, secondhand smoke; FTND, Fagerstrom Test for Nicotine Dependence

^a^strongly disagree 1, disagree 2, neutral, 3, agree 4, strongly agree 5

## Discussion

In previous studies on the factors affecting SHS-related knowledge, assessments were made through relatively ambiguous questions. In addition, few studies have evaluated the factors affecting the degree of knowledge of passive smoking. In the present study, we evaluated the degree of SHS knowledge, sources of information on SHS risk, and SHS- related symptoms in hospital staff.

In the present study, 97.5% of smokers and 100% of non-smokers responded that they were aware of the risks of SHS. However, when asked more specifically using a questionnaire about SHS-related illnesses, 85.5% of non-smokers and 63.8% of smokers answered correctly, which was lower than the subjective perception rate. This implies that, as reported by a previous study, hospital staff have vague knowledge about the risk of SHS [3], but their knowledge about related diseases is not accurate.

A previous study has reported a high level of specific knowledge about SHS at the time of public service advertising for SHS [4]. However, few studies have focused on effective methods of providing information on the risks of SHS. In the present study, there was no correlation between SHS knowledge scores and sources of information on the risks of SHS, except for smoking cessation education (p < .001). TV programs and public service advertising had a high percentage of respondents, but although this method is expected to have the effect of information transmission, it is not enough to convey specific knowledge about SHS risks. This study suggests that smoking cessation education is the most effective way to spread awareness about the risks of SHS, although future studies should continue investigating other effective ways. It has been reported that the effects of exposure to SHS increased with time and the amount of exposure [10]. However, knowledge of SHS and its symptoms has not been studied so far. In this study, knowledge of SHS was found to be higher when there was any exposure symptom or when exposure induced unpleasant feelings. The more likely it is that people are unaware of its risks and the fewer the symptoms they experience, the less likely SHS is to be avoided. Awareness about the consequences of exposing others to SHS may also be lacking. Therefore, it is necessary to strengthen education about SHS risks for people who do not experience the related symptoms.

Among males, in the comparison of smokers and non-smokers, the latter showed statistically significantly higher percentages of correct answers for all questions except for the two related to children. In the comparison of males and females, the latter’s percentage of correct answers on all eight questions related to adults and children was significantly higher than that of males. This suggests that males need more specific education on SHS-related diseases regardless of smoking status.

According to a previous study [8], knowledge of the dangers of SHS exposure for women and children was high among employed Jordanian women with higher education [8], which is consistent with our findings. Another study [4] found that smokers, past smokers, and non-smokers had higher knowledge of SHS, in this order. In the UK, mothers who smoke tend to underestimate the risks of SHS [6]. This may be owing to the lack of knowledge about SHS and may be an attempt to underestimate the risks of SHS owing to cognitive dissonance in smokers [7]. However, unlike in the present study, it has been shown that the knowledge of SHS risks is significantly higher in elderly males than females [3]. These contradictory results may be partly explained by the fact that in the present study, the females were all non-smokers, and most of them were nurses (54.9%) or pharmacists (16.3%).

In this study, SHS exposure was most common in the street. For smokers, smoking areas were the second most common place of SHS exposure, whereas for non-smokers it was public places in general. Smokers would have been exposed to someone else’s cigarette smoke in smoking rooms. Interestingly, smokers reported no exposure to SHS at home, while non-smokers reported that they were exposed to SHS at home. According to 2015 Health Behavior and Chronic Disease Statistics [2], the SHS exposure rate at home was 10.7% for females, which was more than twice as high as that for males (4.2%). This report was similar to the results of our study. This may be owing to the fact that the smoking rate of the spouses of male smokers is very low, which is why smokers feel that there is no SHS exposure at home. In the present study, the exposure rate to SHS in public places, such as restaurants and bars, was 40.3% in non-smokers, while the exposure rate of SHS in the hospital as a workplace, at 4.5%, was very low. This could be a result of the well-established smoking policy in the hospital. According to data from the 2016 Korea National Health and Nutrition Examination Survey, SHS exposure in the workplace steadily declined from 47.3% in 2013 to 17.4% in 2016, and the indoor SHS exposure rate in public places also declined from 57.9% in 2013 to 22.3% [2]. No-smoking areas should be continuously expanded to prevent the harm caused by SHS. However, since both smokers and non-smokers are exposed to SHS on the streets, it is necessary to aggressively establish them as no-smoking zones.

In previous studies, the higher the knowledge of SHS, the higher the tendency to protect oneself from SHS [11–13]. One study reported that males and the elderly were more aware of the risks of SHS [3]. In addition, the higher the awareness of SHS risks, the more likely people will be to advise others around them to quit smoking, leading to active behavior change [3]. However, cultural differences need to be considered. According to a previous study, knowledge of SHS in highly educated Jordanian women was high, but when exposed to SHS, they did not actively engage in action because of the low position of women in Jordan [8]. Another study [14] found that the higher the knowledge of SHS among Asian Americans who immigrated to the United States, the more active their efforts to avoid SHS, and the longer they had stayed in the United States, the more likely they were to exhibit the aggressive behavior required to avoid smoking if exposed to SHS. In Asian societies, where the culture emphasizes harmony with others, it is difficult to outright ask someone to stop smoking even when the knowledge about SHS is high. Therefore, in this study, it is considered that there is no relationship between knowledge of SHS and actively preventing others from smoking.

The limitation of this study is that as it was conducted at a university hospital, our results may not be generalizable to a specialty setting or other populations. In addition, the questionnaire response was obtained by securing a sample size that satisfied the 95% confidence interval with an error margin of 5% for the total number of employees but recall bias could have affected the result because the study was conducted with a self-administered questionnaire. Although this was an anonymous survey, it is thought that smoking females would have hesitated to admit to smoking because of social perceptions. Nevertheless, the strength of this study is that, to the best of our knowledge, it is the first on SHS conducted among hospital staff, and it assessed the relevant knowledge by dividing it into detailed SHS risk items.

## Conclusion

In conclusion, this study suggests that SHS knowledge is high among females, when hospital staff are educated about SHS risks, and when they have experienced any SHS exposure-related symptoms or felt uncomfortable when exposed to SHS. Therefore, to increase the knowledge of SHS, there is a need to actively impart education regarding its risks.

## Supporting information

S1 File Questionnaire in Korean. (DOCX)

S2 File Questionnaire in English. (DOCX)

S1 Dataset Raw data. (XLSX)
