# Supplementary material for: Secondhand smoke knowledge, sources of information, and associated factors among hospital staff
Source: PLoS One. 2019 Jan 22;14(1):e0210981. doi: 10.1371/journal.pone.0210981 (PMC6342318; doi:10.1371/journal.pone.0210981)
Supplement: S1 File — (DOCX) [file pone.0210981.s001.docx]

**Questionnaire in Korean**

| 안녕하십니까? 가정의학과 전공의 이새롬입니다. 양산부산대학교 병원 직원을 대상으로 간접흡연에 대한 지식과 흡연 및 금연 태도에 관한 설문 조사를 통해 논문을 쓰고자 합니다. 바쁘신 가운데 시간내어 주시어 설문에 응해 주셔서 감사합니다. |
| --- |

| 나이 | 만 세 | 성별 | ①남 ②여 |
| --- | --- | --- | --- |

**1. 현재 동거하고 있는 사람은 몇 명(본인제외) 입니까?** 명

**2-1. 현재 배우자와 동거하고 있습니까?** ➀예 ➁아니요

**2-2. 현재 자녀와 동거하고 있습니까?** ➀예 ➁아니요

**3. 학교를 어디까지 다녔습니까?**

①무학 ②초등학교 ③중학교 ④고등학교 ⑥대학교 ⑦대학원

**4. 현재 진단 받은 질병이 있으십니까?(진단받은 질병 모두 표시해주세요)**

①심장질환 ②호흡문제, 폐질환, 천식 ③뇌졸중 ④당뇨병 ⑤고혈압 ⑥암 ⑦기타

**5. 양산부산 대학병원에 근무하는 직종이 무엇입니까?**

①간호직 ②보건직 ③약무직 ④의사직 ⑤행정직/기술직 ⑥기타

**6. 양산부산 대학병원에 입사한 년도는 언제입니까?**  년

**7. 최근 1년간 음주를 했습니까?**

➀예(7-1 항목부터 답해주세요 ) ➁아니요(II 항목부터 답해주세요)

**7-1. 일주일동안 음주 횟수와 음주했을 경우 음주량이 어떻게 됩니까?**

주 회, 소주 잔/ 맥주 잔

**II. 설문조사- 흡연자 평가**

**1.담배를 피웁니까?**

①피운다

③과거(6개월 이전)에는 피웠으나 현재 피우지 않음.

④피운적 없음(->III 항목부터 답해주세요)

**2.하루 담배 피우는 개수, 담배를 피운지는 얼마나 됩니까?**  하루 개피, 년

**3.처음 흡연시작 연령은 언제였습니까?** 세

**4-1. 아침에 일어나서 얼마 만에 첫 담배를 피우십니까?**

①5분 이내 ②6분~30분 사이 ③ 31분~1시간 사이 ④1시간 이후

**4-2. 금연구역(도서관, 극장, 병원 등)에서 담배를 참기가 어렵습니까?**

①예 ② 아니오

**4-3. 하루 중 담배 맛이 가장 좋은 때는 언제입니까?**

①아침 첫 담배 ②그 외의 담배

**4-4. 하루에 담배를 보통 몇 개비나 피우십니까?**

① 31개비 이상 ②1~30개비 ③ 11~20개비 ④ 10개비 이하

**4-5. 오후와 저녁시간보다 오전 중에 담배를 더 자주 피우십니까?**

①예 ② 아니오

**4-6. 몸이 아파 하루 종일 누워있을 때에도 담배를 피우십니까?**

① 예 ②아니오

**5. 담배를 끊고자 금연을 시도한 적이 있습니까? 있다면 몇회입니까?**

①예 (총 회, 가장 오랫동안 금연시도기간: 개월, 일) ②아니요

**6. 앞으로 담배를 끊을 계획이 있습니까?**

①1달 이내에 금연할 계획이다. ②6개월내에 금연할 생각이 없다.

③6개월 이내는 아니지만 언젠가는 금연할 생각이 있다. ④이전에 담배를 피웠지만 금연한지 6개월 미만이다.

⑤이전에 담배를 피웠지만 금연한지 6개월 이상이다.

**7. 담배를 끊으려고 했던 가장 큰 이유는 무엇 때문입니까?(하나만 체크하세요)**

①건강이 나빠져서 ②현재 건강은 이상 없지만 향후 건강에 대한 염려로

③가족의 건강을 위해서 ④주변사람들에게 피해를 주지 않기 위해

⑤주위 사람들의 권유로 ⑥담뱃값이 부담돼서

⑦금연공익광고를 보고 ⑧사회생활이 불편해서(흡연장소가 없어서)

⑦기타 ⑨비해당(금연을 시도한 적 없음)

**8. 주로 담배를 어디서 피웁니까?(주로 피우는 장소 2군데까지 표시해 주십시오)**

①집-거실, 방안 ②집-베란다, 복도

③병원건물 주변 ④병원건물 내(화장실, 하늘공원, 비상계단, 옥상)

⑥공공장소(식당, 술집, 카폐, 화장실) ⑦길거리

⑧지정된 흡연 장소 ⑨기타

**III. 설문조사-간접흡연**

**1. 타인의 담배연기에 노출되는 것(간접흡연)의 폐해에 대해 알고 있습니까?**

①알고 있다. ②모른다.

**2-1. 타인의 담배연기에 노출되는 것(간접흡연)이 폐암을 일으킬 수 있다.**

①매우 그렇다. ②그렇다. ③보통이다. ④그렇지 않다. ⑤매우그렇지 않다.

**2-2. 간접흡연이 심장질환을 일으킬수 있다.**

①매우 그렇다. ②그렇다. ③보통이다. ④그렇지 않다. ⑤매우그렇지 않다.

**2-3. 간접흡연이 두뇌 할동에 영향을 미친다.**

①매우 그렇다. ②그렇다. ③보통이다. ④그렇지 않다. ⑤매우그렇지 않다.

**2-4. 임산부의 간접흡연은 저체중아를 출산하게 한다.**

①매우 그렇다. ②그렇다. ③보통이다. ④그렇지 않다. ⑤매우그렇지 않다.

**2-5. 간접흡연은 어린아이의 중이염을 일으킬 수 있다.**

①매우 그렇다. ②그렇다. ③보통이다. ④그렇지 않다. ⑤매우그렇지 않다.

**2-6. 간접흡연은 어린아이의 심장마비를 일으킬 수 있다.**

①매우 그렇다. ②그렇다. ③보통이다. ④그렇지 않다. ⑤매우그렇지 않다.

**2-7. 간접흡연은 어린아이의 알러지와 관련이 있다.**

①매우 그렇다. ②그렇다. ③보통이다. ④그렇지 않다. ⑤매우그렇지 않다.

**2-8. 간접흡연은 어린아이의 천식과 관련이 있다.**

①매우 그렇다. ②그렇다. ③보통이다. ④그렇지 않다. ⑤매우그렇지 않다.

**3. 간접흡연에 대해 알게된경로가 어떻게 됩니까?(pilot study 참조)**

**4. 간접흡연에 노출된 적이 있습니까?**

①있다 ②없다.(8 번으로)

**5. 간접흡연에 노출되는 장소는 어디입니까?(가장 많이 노출된 곳 2군데 까지 표시하세요)**

①집-거실, 방안 ②집-베란다, 복도

③병원건물 주변 ④병원건물 내(화장실, 하늘공원, 비상계단, 옥상)

⑥공공장소(식당, 술집, 카폐, 화장실) ⑦길거리

⑧지정된 흡연 장소 ⑨기타

**6. 간접흡연 되었을 때 어떤 기분이 드십니까?**

➀기분이 좋다. ➁불편하지 않다. ➂약간 불편 하다. ➃많이 불편하다. ➄고통스럽다.

**7. 다음중 간접흡연으로 인해 느낀 증상이 있습니까?(모두 표시해주세요)**

①눈이나 코의 자극 증상 ②호흡기 증상

③흉통 ④소아의 호흡기 증상

⑤기타 ⑥느껴본 적 없다.

**8. 간접흡연에 노출되었을 경우 어떻게 합니까?**

①담배를 꺼줄 것을 요청한다. ②자리를 피한다. ③아무것도 하지 않는다.

➃같이 담배를 피운다. ➄기타

**9. 공공장소에서 금연구역을 설정하여 담배를 못 피우게 하는 것에 대해 어떻게 생각하십니까?**

①담배 피우는 권리를 무시한 부당한 처사이다.

②법적/강제적 규제보다는 각 개인의 개인적인 양심에 맡기는 것이 더 좋다.

③법적/ 강제적으로라도 금연구역을 학대하여 보다 더 엄격하게 규제해야한다.
