# Supplementary material for: Secondhand smoke knowledge, sources of information, and associated factors among hospital staff
Source: PLoS One. 2019 Jan 22;14(1):e0210981. doi: 10.1371/journal.pone.0210981 (PMC6342318; doi:10.1371/journal.pone.0210981)
Supplement: S2 File — (DOCX) [file pone.0210981.s002.docx]

**Questionnaire in English**

Part I. Basal characteristic

- 1. What is your age? years
  2. What is your gender?

1. Female b. Male
   1. How many people are you living with?
   2. Are you currently living with your child?

a. Yes b. No

- 1. Are you currently living with your spouse?

a. Yes b. No

3. What is the highest level of formal education you have completed?

1. Elementary school b. Middle school c. High school d. University

e. Graduate school

4. What is your occupation?

1. Nurses b. Health care staffs c. Pharmacists d. Physicians

e. Administrative staffs f. Manual workers

5. How long you have been working in PNYUH? years

6. Have you ever been diagnosed with any of the following?

a. Heart disease b. Respiratory disease c. Stroke d. Diabetes mellitus

e. Hypertension f. cancer g. etc.

7-1. Did you drink currently (in 1 year)?

1. Yes b. No

7-2. How many glasses of alcohol do you drinking?

Glasses SOJU/ Glasses BEER

7-3. How often do you drink alcohol per week? Days per week

Part II. Smoking

1. Do you currently use tobacco?

a. Yes, on a regular use

b. Not anymore, I quit (more than 6 months)

c. No, I have never used tobacco

2. How many cigarettes did you smoke per day? cigarettes per day years

3. Please write age of initiation year of tobacco use. years

4-1. How soon after you wake up do you smoke your first cigarette?

1. With 5 minutes
2. 6-30 minutes
3. 31-60 minutes
4. After 60 minutes

4-2. Do you find it difficult to refrain from smoking in places where it is forbidden, e.g., in church, at the library, in the cinema, etc.?

1. Yes
2. No

4-3. Which cigarettes/day do you smoke?

1. 10 or less
2. 11-20
3. 21-30

4-4. Do you smoke more frequently during the first hours after waking than during the rest of the day?

1. Yes
2. No

4-5. Do you smoke if you are so ill that you are in bed most of the day?

1. Yes
2. No

5. Have you tried to stop smoking, and how many you tried?

a. Yes

b. No

6. Do you have intended to quit tobacco use?

a. Planning to quit the next 30 days

b. Planning to quit but not in 6 months

c. Have no intention of quitting in six months

d. Have been quit smoking (less than 6 months)

e. Have been quit smoking (more than 6 six months)

7. What is the one most important reason you want quit tobacco? (Check one)

a. Have a disease

b. For health care

c. For family health

e. Not to harm people around me

f. By acquaintance propose

g. Financial burden (buying cigarettes)

1. Public service announcement
2. Uncomfortable to find out smoking place
3. Have no intention of quitting smoke

8. Where did you usually smoke? (check two)

a. At home-living room, in room

b. At home-outside room

c. Around the hospital

d. In the hospital

e. Public places

f. Street corners

g. At smoking places

Part III. Second hand smoking

1. Did you know SHS health risk?
2. Yes b. No
3. On a scale where 5 represents strongly agree and 1 represents strongly disagree how would you rate each of the following statements?
   1. SHS causes lung cancer.
4. Strongly agree b. Agree c. Neutral d. Disagree e. Strongly disagree
   1. SHS causes heart disease.
5. Strongly agree b. Agree c. Neutral d. Disagree e. Strongly disagree
   1. SHS is associated with cognitive deficits.
6. Strongly agree b. Agree c. Neutral d. Disagree e. Strongly disagree
   1. SHS causes low birth weight.
7. Strongly agree b. Agree c. Neutral d. Disagree e. Strongly disagree
   1. SHS causes ear infection in children.
8. Strongly agree b. Agree c. Neutral d. Disagree e. Strongly disagree
   1. SHS causes heart attack to children.
9. Strongly agree b. Agree c. Neutral d. Disagree e. Strongly disagree
   1. SHS is associated with allergies in children.
10. Strongly agree b. Agree c. Neutral d. Disagree e. Strongly disagree
    1. SHS is associated with asthma in children.
11. Strongly agree b. Agree c. Neutral d. Disagree e. Strongly disagree
12. How did you get to know SHS health risk?
13. Newspapers
14. TV programs
15. Public service announcement
16. Smoking cessation education
17. Acquaintance
18. Have you been exposed to SHS by any chance?
19. Yes b. No
20. Where did you usually exposed to SHS?

a. At home-living room, in room

b. At home-out side room

c. Around the hospital

d. In the hospital

e. Public places

f. Street corners

1. How do you feel when exposed to SHS?
2. Feels good
3. No uncomfortable feeling
4. Little bit uncomfortable
5. Very uncomfortable
6. Painful
7. Did you have any symptoms when exposed to SHS? (check all)
8. Irritation of the nose and the eyes
9. Respiratory symptom
10. Chest discomfort
11. Child respiratory
12. Any symptom
13. How do you cope with exposure to secondhand smoke?
14. I will ask them to refine from smoking
15. I will move a way to avoid SHS
16. Do nothing
17. Smoke together
18. What do you think about stop smoking public places?
19. It is unfair to ignore the right to smoke.
20. It is better to leave it to each individual's personal conscience than to compulsory regulations.
21. Even if it is forced to abuse the smoking area, it should be regulated more strictly.
